# Opposition to State Examiners in Ohio

**Published:** 1895-05

**Authors:** 


### EDITORIAL.

THE June number of the JOURNAL will contain an exhaustive review of all new publications of interest to the veterinarian and student of comparative medicine.

STATE BOARDS OF VETERINARY MEDICAL EXAMINERS.—The actions of veterinarians in Ohio refusing to conform to the requirements of the State Board of Veterinary Medical Examiners need surprise none who are conversant with the low standards existing at some of the veterinary colleges of North America; nor need it cause any alarm to these Boards or the profession at large. These graduates, when once registered and in practice, will prove to be the strongest advocates of this well-devised safeguard for the people of any State. They will fully appreciate the fact that diplomas—more properly speaking, certificates of study—have relative degrees of value that vary exceedingly, and, like one dozen pieces of silk, they are all silk or all diplomas, but each of a different quality and representing different types of material and skill, different requirements of study, different masters skilled in the art of instruction. None know these material facts better than the schools themselves, and those who aim and strive to uphold and create a higher standard of educational requirements will be found supporting and sustaining these laws, destined to win for the profession in every State where they exist that better recognition and respect without which no vocation can command its rightful place among the people. Let the good work go on; let the lines be drawn; let every member jealous of the good name of his profession watch closely every movement in this direction.

